# Genetic diversity analysis of brown marmorated stink bug, *Halyomorpha halys* based on mitochondrial COI and COII haplotypes

**DOI:** 10.1186/s12863-021-00961-8

**Published:** 2021-02-15

**Authors:** Juncong Yan, Chandan Pal, Diane Anderson, Gábor Vétek, Péter Farkas, Allan Burne, Qing-Hai Fan, Jinping Zhang, Disna N. Gunawardana, Rebijith Kayattukandy Balan, Sherly George, Dongmei Li

**Affiliations:** 1 Plant Health and Environment Laboratory, Ministry for Primary Industries, PO Box 2095, Auckland, 1140 New Zealand; 2 Plant Health and Environment Laboratory, Ministry for Primary Industries, PO Box 14018, Christchurch, 8544 New Zealand; 3 Department of Entomology, Szent István University, Villányi út 29-43, Budapest, H-1118 Hungary; 4 Biosecurity Science and Risk Assessment, Ministry for Primary Industries, Wellington, New Zealand; 5 MARA-CABI Joint Laboratory for Bio-safety, Institute of Plant Protection, Chinese Academy of Agricultural Sciences, No. 2 Yuanmingyuan West Road, Beijing, 100193 People’s Republic of China

**Keywords:** BMSB, Invasion, Mitochondrial DNA, Nucleotide diversity, Haplotype diversity, Pathway

## Abstract

**Background:**

In the past decade, the brown marmorated stink bug (BMSB), *Halyomorpha halys* (Hemiptera: Pentatomidae) has caused extensive damage to global agriculture. As a high-risk pest for many countries, including New Zealand, it is important to explore its genetic diversity to enhance our knowledge and devise management strategies for BMSB populations. In this study, two mitochondrial genes, Cytochrome c oxidase I (COI) and Cytochrome c oxidase II (COII) were used to explore the genetic diversity among 463 BMSB individuals collected from 12 countries.

**Result:**

In total, 51 COI and 29 COII haplotypes of BMSB were found, which formed 59 combined haplotypes (5 reported and 54 novel). Of these, H1h1 was the predominant haplotype. The haplotype diversity (*Hd*) and nucleotide diversity (***π***) were high while the neutrality (Fu’s Fs) values were negative for the BMSB populations in the native countries, China, and Japan. For the BMSB populations from the invaded countries, the Fu’s Fs values were negative for populations from Chile, Georgia, Hungary, Italy, Romania, Turkey, and USA, indicating that those populations are under demographic expansion. In comparison, the Fu’s Fs values were positive for the populations from Austria, Serbia, and Slovenia, revealing a potential population bottleneck. Analysis of molecular variance (AMOVA) suggested that significant genetic difference exists among the BMSB populations from China, Japan, and the invasive countries.

**Conclusion:**

This study revealed that the haplotype diversity of the BMSB populations was high in those two studied countries where BMSB is native to (China and Japan) but low in those countries which have been invaded by the species. The analysis indicated that multiple invasions of BMSB occurred in Europe and the USA. The study also revealed three ancestral lines and most of the novel haplotypes were evolved from them. Moreover, we observed two genetic clusters in the invasive populations that are formed during different invasion events. Our study provided a comprehensive overview on the global haplotypes distribution thus expanding the existing knowledge on BMSB genetic diversity that potentially could play an important role in formulating feasible pest management strategies.

**Supplementary Information:**

The online version contains supplementary material available at 10.1186/s12863-021-00961-8.

## Background

Brown marmorated stink bug (BMSB), *Halyomorpha halys* (Stål, 1855) (Hemiptera: Pentatomidae) is a highly polyphagous insect, which feeds on more than 300 host plants [1, 2]. BMSB has been causing extensive damage to a wide variety of agricultural crops and poses a global economic threat for agricultural and horticultural industry [3, 4]. As per 2010 reports, the economic losses caused by BMSB valued more than 37 million USD in North America [5]. BMSB is capable of long-distance flight as well as local walking dispersal during the growing season [6, 7]. They are also considered nuisance pests as adults search for human-made structures to overwinter and discharge an unpleasant and long-lasting odor once disturbed [8].

BMSB is native to China (including Taiwan), Japan and the Korean peninsula [9–11] and has invaded 30 countries [12] including most of the states in the USA [4, 5], Canada [13], many countries in Europe [13–20] and Chile [21]. Climate modelling predicted that New Zealand at high risk for BMSB establishment should a successful invasion occur [22, 23]. The climatic suitability of this species is wide, ranging from 30 to 50 degrees of latitude, with an annual mean temperature range between 10 °C and 30 °C (NOAA-CIRES Climate Diagnostics Center, Boulder, Colorado). It covers wide geographical regions including northern Europe, north eastern part of North America, southern Australia, and the North Island of New Zealand [22, 23]. BMSB has increasingly been intercepted at the border and post-border scenarios due to the rise of international travel and trade [24] since 2005 when BMSB was first intercepted at the border of New Zealand [25]. BMSB is considered as “high risk” organism, and there were 1620 recorded interceptions of BMSB at the border of New Zealand since 2005 (accessed in June 2020) [25]. As agricultural exports comprise a significant proportion of the New Zealand Gross Domestic Product (GDP), the establishment of the pest would be highly detrimental to the country. The study by New Zealand Institute of Economic Research (NZIER) predicts a worst-case scenario of 3.6 billion New Zealand dollars in agricultural losses by 2038 if BMSB successfully establishes in New Zealand [26]. Therefore, it is imperative to study the genetic diversity of BMSB to trace the origin of captured individuals to better manage the border biosecurity risks and predict the pathways for trade.

Mitochondrial DNA (mtDNA) sequence analysis is one of the most widely used methods to examine genetic diversity and determine the locality of origin of an invasive species [27], and has been applied in a number of studies for BMSB [13, 20, 28–30]. Previous studies on BMSB populations from Asia and the USA have sequenced the mitochondrial Cytochrome Oxidase II (COII) and the ribosomal 12S genes to trace the origin of the invading BMSB populations in the USA [27]. Additional studies, focusing on BMSB populations in Europe, used Cytochrome Oxidase I (COI) gene alone [13], or in combination with the COII gene [20, 30], and revealed additional information on the genetic diversity of BMSB [13, 31]. To expand the knowledge on the genetic diversity of BMSB populations around the world, we collected BMSB adult specimens from different geographical locations, including the countries where BMSB is native to, China and Japan and the countries which have been invaded by the pest, such as the USA, Chile, and several European countries. In the current study, two mitochondrial barcoding genes, COI and COII, were sequenced from 463 BMSB specimens collected from 43 regions/provinces in 12 countries (2 native and 10 invaded) across four different continents to develop new insights into BMSB genetic diversity and their potential pathways of invasion.

## Results

### COI and COII haplotypes in the BMSB populations

A total of 441 COI sequences (657 bp each) were obtained from 463 BMSB individuals collected from the 12 countries (Additional file 1). We identified 51 haplotypes using COI, consisting of 36 newly identified and 15 previously reported haplotypes (Additional file 2). For the sequences sharing 100% identity in the same region (657 bp) with those previously reported, the same haplotype names were given while new names were given accordingly for the rest of the sequences obtained in this study. All the new haplotypes identified were confirmed by BLASTn search [32]. The result showed that all the new haplotypes were unique in the COI region (657 bp). Further analysis showed that the haplotype N22 shared the same sequences with two shorter reference sequences, KY710432 (651 bp) and KY710450 (648 bp). However, it is not clear whether the missing bases from the two reference sequences are the same or different from the sequence we obtained, thus the sequence was considered as a new haplotype. The analysis also indicated that it might not be accurate to assign the same haplotype name for the sequence with different length.

There are three Barcode Index Numbers (BINs) for the COI sequences of BMSB specimens submitted in Barcode of Life Data System (BOLD) [33]: BIN AAM9563 (containing 682 sequences), ADT6053 (one sequence) and AAK5312 (six sequences). Further comparison with those COI sequences in BOLD showed that all the sequences obtained in this study belong to BIN AAM9563, with over 98% identity. In contrast, the sequences shared over 94 and 82% identity to the sequences of two other BINs, ADT6053 and AAK5312, respectively.

A total of 450 COII sequences (518 bp) were obtained from the 463 BMSB individuals (Additional file 1), which formed 29 haplotypes including 20 novel and 9 previously reported (Additional file 2). BLASTn search [32] showed that the new haplotypes did not share any identical sequences in the 518 bp overlap COII region with that of the previous reported sequences.

**Fig. 1:**
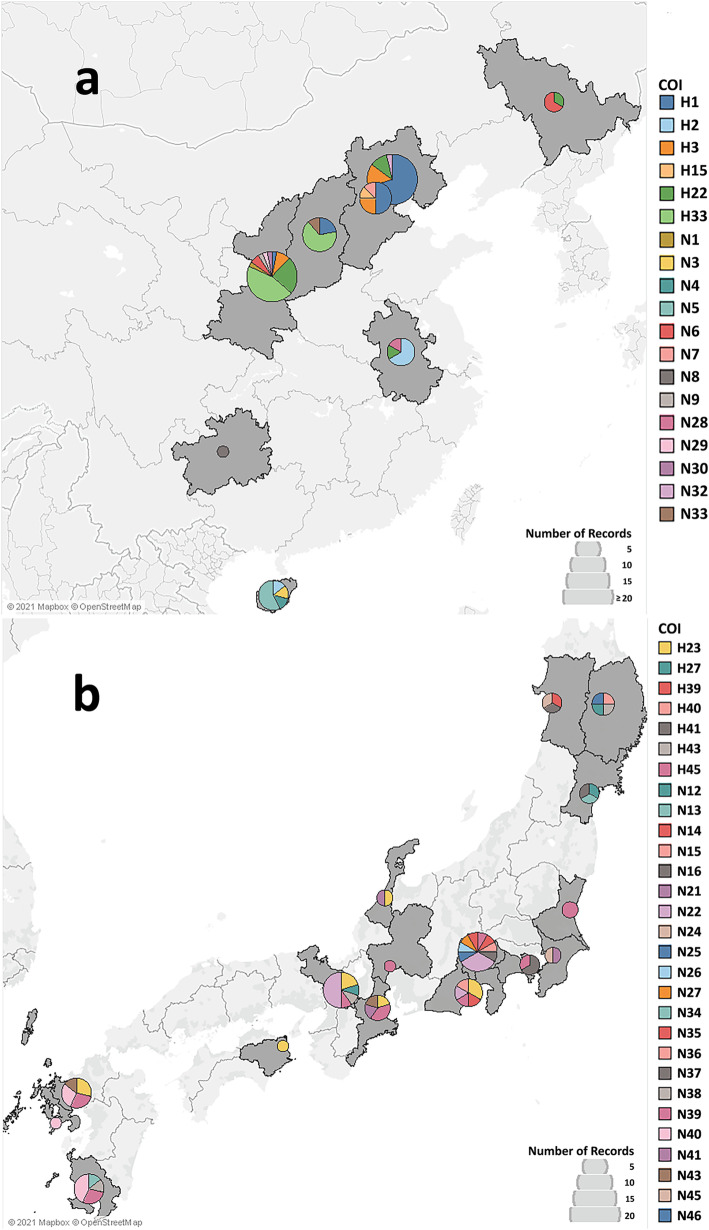
Geographical distribution of mtDNA COI haplotypes (657 bp fragment of the COI) of BMSB in native countries - (**a**) China, and (**b**) Japan. Provinces where BMSB was collected from are shaded in grey. The size of pie is proportional to the frequency of haplotypes. Each colour represents a different haplotype. The figure was generated using Tableau 2019 (https://www.tableau.com/) [34]

**Fig. 2:**
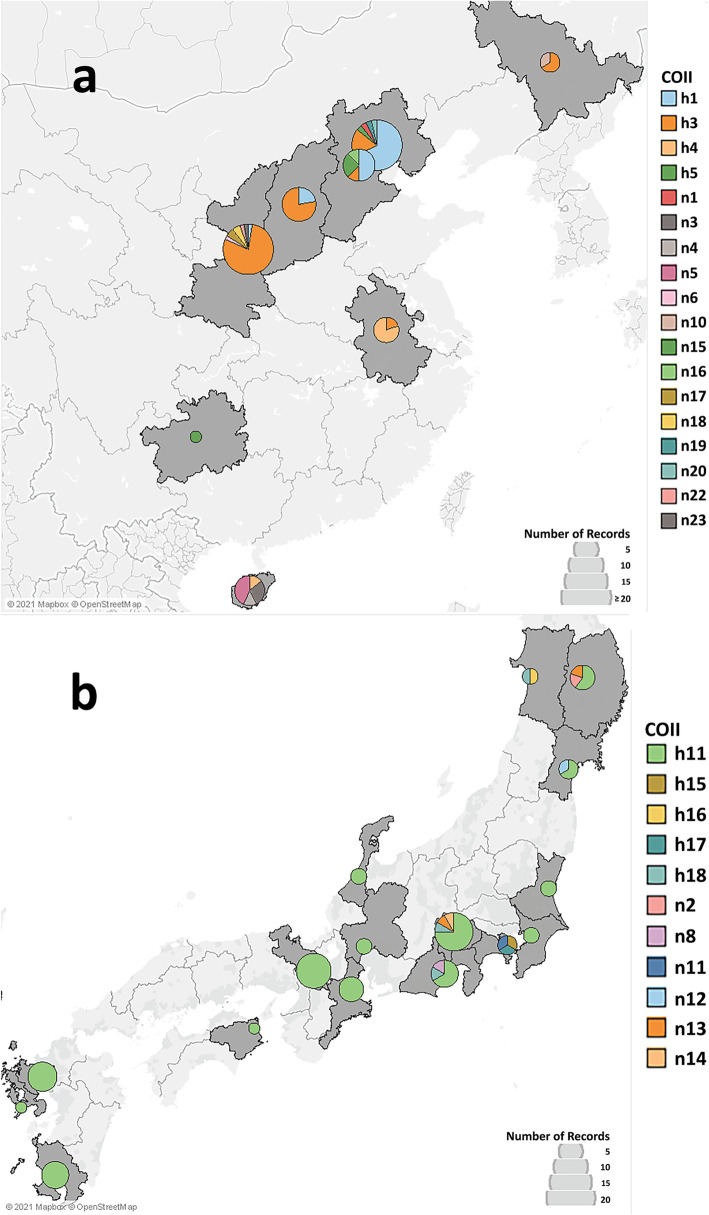
Geographical distribution of mtDNA COII haplotypes (518 bp fragment of the COII gene) of BMSB in native countries in the current study – (**a**) China, and (**b**) Japan. Regions where BMSB was collected from are shaded in grey. The size of pie is proportional to the frequency of haplotypes. Each colour represents a different haplotype. The figure was generated using Tableau 2019 (https://www.tableau.com/) [34]

The geographical distribution of the identified COI and COII haplotypes are shown in Figs. 1 and 2. Of the identified haplotypes, H1 (61.9% of the total individuals) and h1 (61.7% of the total individuals) were predominant for COI and COII, respectively, and were detected in all the countries studied except Japan (Table 1 and Additional file 2). Haplotypes H3 (7% of the total individuals) and h3 (16% of the total individuals) were the second most predominant haplotypes detected in China, Austria, Chile, Hungary, Italy, Serbia, and Slovenia. In addition, haplotypes H8 and H48 for COI were only detected in Austria. The newly identified haplotypes were mainly observed in the native countries (Fig. 1a for China and Fig. 1b for Japan) except N47 in Slovenia (Additional file 2). All the novel COI and COII haplotypes identified were detected from the two native countries, China (Figs. 1 and 2a) and Japan (Figs. 1 and 2b).

**Table 1:** Percentage (%) of the dominant mtDNA COI and COII haplotypes of *H. halys* detected in the studied countries*.* The COI haplotypes are named in uppercase letters. The haplotype names with a prefix ‘H’ represent the previously reported haplotypes while those with ‘N’ are the newly detected haplotypes identified in this study. The percentages of the individuals for each dominant haplotype in the country are listed in the table

Country	COI								COII		
	H1	H3	H22	H33	H23	H45	N22	N40	h1	h3	h11
**China**	28	10	14	22	–	–	–	–	26	46	–
**Japan**	–	–	–	–	16	16	14	9	–	–	81
**Austria**	25	50	–	–	–	–	–	–	20	67	–
**Serbia**	50	50	–	–	–	–	–	–	44	56	–
**Slovenia**	69	25	–	–	–	–	–	–	67	33	–
**Chile**	97	3	–	–	–	–	–	–	97	3	–
**Georgia**	100	–	–	–	–	–	–	–	100	–	–
**Hungary**	93	5	–	–	–	–	–	–	94	4	–
**Italy**	96	4	–	–	–	–	–	–	93	7	–
**Romania**	100	–	–	–	–	–	–	–	100	–	–
**Turkey**	100	–	–	–	–	–	–	–	100	–	–
**USA**	100	–	–	–	–	–	–	–	100	–	–

Overall, high haplotype diversity was observed in China. The main haplotypes from China were H1, H33, H22, H3 for COI and h3 and h1 for COII (Table 1). The predominant haplotypes from Japan were H23, H45, N22, N40 for COI and h11 for COII (Table 1). Outside of the native regions, low haplotype diversity was observed, and H1, H3 for COI, h1, h3 for COII were the main haplotypes detected in those countries. Only one haplotype of each (H1 and h1) was detected in Georgia, Romania, Turkey and the USA (Table 1).

### COI-COII combined haplotypes of the BMSB populations

**Fig. 3:**
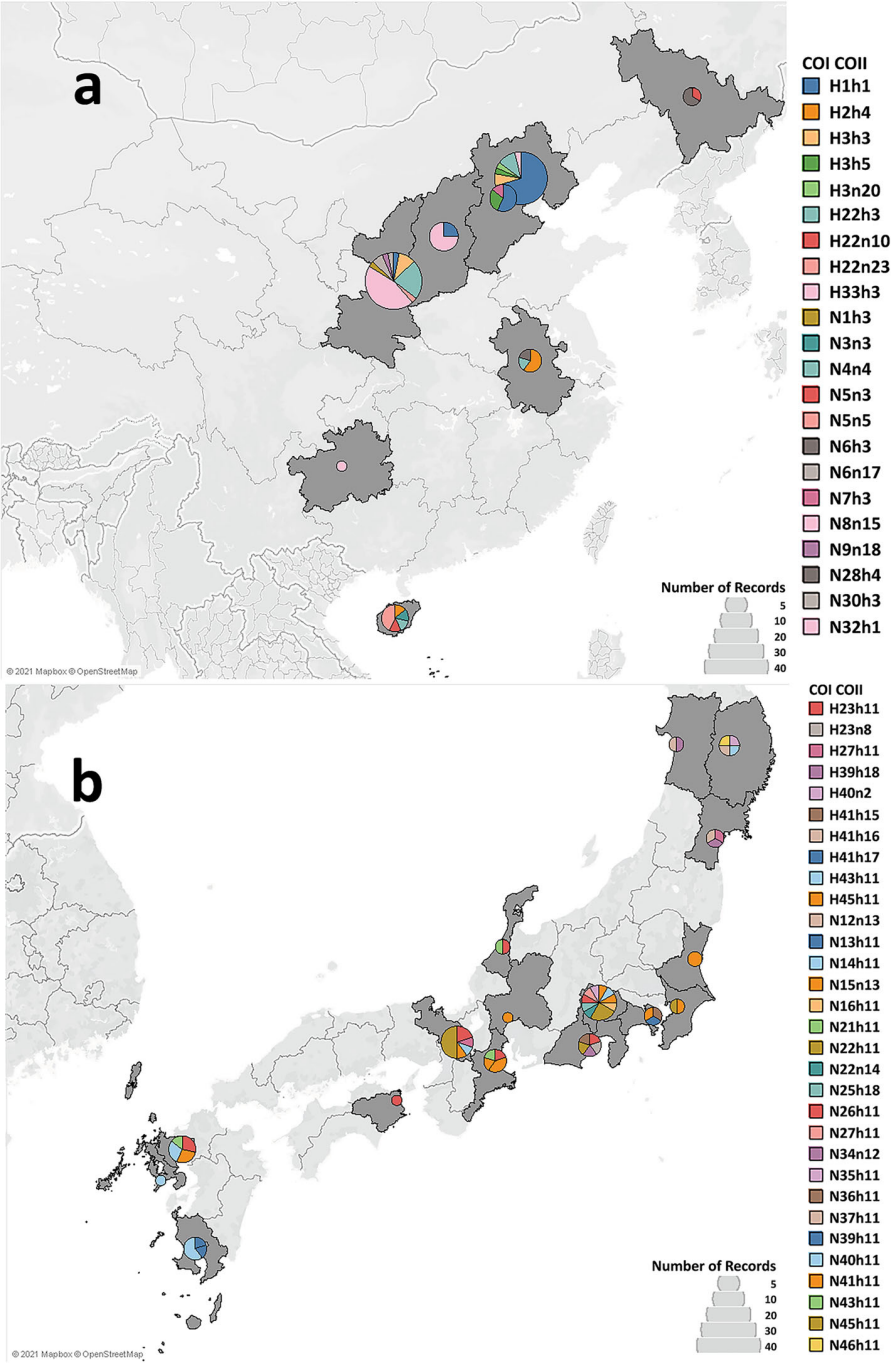
Geographical distribution of mtDNA COICOII haplotypes (1175 bp fragment) of BMSB in native countries in the current study, (**a**) China, and (**b**) Japan. Provinces where BMSB was collected from are shaded in grey. COI-COII combined haplotypes were formed by combing COI and COII haplotypes. The size of pie is proportional to the frequency of haplotypes. Each colour represents a different haplotype. The figure was generated using Tableau 2019 (https://www.tableau.com/) [34]

In total, 428 individuals were identified with both COI and COII sequences (Additional file 1), and thus used for COI-COII combined haplotype analysis. The combined COI-COII haplotype analysis produced 59 haplotypes, in which only five were previously reported and 54 were novel (Additional file 2). All these newly identified haplotypes were detected in China and Japan except a single haplotype in Slovenia (N47h3). The predominant haplotype H1h1 (62.6%) was observed in all the countries except Japan (Additional file 2). The geographical distribution of the identified COI-COII combined haplotypes is shown in Fig. 3. In the native countries of BMSB, high haplotype diversity was observed with 24 haplotypes in China (Fig. 3a) and 32 in Japan (Fig. 3b), with no haplotypes shared between the two countries (Additional file 2 and Fig. 3). In comparison, out of the 32 haplotypes identified in Japan, 31 were uniquely detected in Japan, and one haplotype, H41h15 was shared with an individual from Hungary (Additional file 2). Similarly, 22 out of 24 haplotypes detected in China were unique, and two haplotypes (H1h1 and H3h3) were also predominantly shared with the BMSB samples from those invaded countries (Additional file 2). In the invaded countries, H1h1 was the predominant haplotype, identified in more than 90% of the studied samples from most of the BMSB-invaded countries, including Chile, Georgia, Hungary, Italy, Romania, Turkey and the USA (Additional file 2).

### Population genetic analysis based on the combined haplotypes of COI and COII

Japan and China had the highest haplotype diversity (*Hd*), with *Hd* values of 0.942 and 0.858, and nucleotide diversity (***π)*** values of 0.00238 and 0.00327, respectively (Table 2). Outside of the native regions of BMSB, the highest haplotype diversity was observed in Austria (*Hd* = 0.686, ***π*** = 0.00206), Serbia (*Hd* = 0.556, ***π*** = 0.00095) and Slovenia (*Hd* = 0.514, ***π*** = 0.00115). In contrast, little to no haplotype diversity was observed in the BMSB samples collected from Chile, Georgia, Hungary, Italy, Romania, Turkey and the USA. Therefore, two genetic groups were defined based on the *Hd* values obtained from the haplotype analysis: group A (Chile, Georgia, Hungary, Italy, Romania, Turkey and the USA) and group B (Austria, Serbia and Slovenia). It is noteworthy that in Hungary five sampling sites were studied, of which at two sites no haplotype diversity was observed, while the other three sites showed variable diversity with an *Hd* value from 0.038 to 0.5 and a ***π*** value from 0.00085 to 0.0017036, with an overall *Hd* value of 0.107 and a ***π*** value of 0.00028. This indicates that the invasion of BMSB in Hungary may have come from genetically distinct populations.

**Table 2:** The sample information and mtDNA diversity. The total sample size (N) for each province and country is listed. The number of haplotypes (*Hn*), haplotype diversity (*Hd*) and nucleotide diversity (*π*) were calculated based on the combined haplotypes of COI and COII. Two genetic groups were identified based on the COI and COII diversity from invaded populations. The genetic group A comprises Chile, Georgia, Hungary, Italy, Romania, Turkey, and the USA. The genetic group B comprises Austria, Serbia, and Slovenia. The analysis was conducted for each population, the populations from each country and each genetic group. Asterisk (*) represents statistically significant difference (*p* < 0.02)

Country	Province	***N***	***Hn***	***Hd***	***π***	Fu’s Fs
**China**	Anhui	5	3	0.7	0.00102	
	Beijing	27	7	0.553	0.00113	
	Hainan	7	5	0.857	0.00284	
	Hebei	8	4	0.75	0.00225	
	Jiling	3	2	0.667	0.00227	
	Shannxi	31	9	0.751	0.00205	
	Shanxi	8	2	0.429	0.00182	
	Guizhou	1	1	NA	NA	
	Combined	90	24	0.858	0.00327	−7.852*
**Japan**	Akita	2	2	1	0.0017	
	Chiba	2	2	1	0.0017	
	Gifu	1	1	NA	NA	
	Ibaraki	2	1	0	0	
	Ishikawa	2	2	1	0.00255	
	Iwate	4	4	1	0.00426	
	Kagoshima	5	3	0.7	0.00102	
	Kanagawa	3	3	1	0.00454	
	Kyoto	10	5	0.756	0.00127	
	Mie	5	4	0.9	0.00136	
	Miyagi	3	3	1	0.0034	
	Nagasaki	1	1	NA	NA	
	Saga	7	4	0.857	0.00105	
	Shizuoka	5	5	1	0.00221	
	Tokushima	1	1	NA	NA	
	Yamanashi	12	10	0.955	0.00253	
	Combined	65	32	0.942	0.00238	−29.707*
**Serbia**	Senta	9	2	0.556	0.00095	2.302
**Slovenia**	Ljubljana	15	3	0.514	0.00115	1.626
**Austria**	Vienna	15	4	0.686	0.00206	1.84
**Turkey**	Arhavi	11	1	0	0	0
**the USA**	Maryland	15	1	0	0	
	West Virginia	9	1	0	0	
	Combined	24	1	0	0	0
**Georgia**	Eki	28	1	0	0	
	Samegrelo	3	1	0	0	
	Combined	31	1	0	0	0
**Hungary**	Budapest	61	3	0.038	0	
	Debrecen	10	1	0	0	
	Pécs	6	1	0	0.00085	
	Szeged	2	2	0.5	0.00136	
	Szombathely	11	3	0.345	0	
	Combined	90	3	0.107	0.00028	−0.195*
**Italy**	Codroipo (UD)	4	1	0	0	
	Mantova	2	1	0	NA	
	Pozzuolo del Friuli (UD)	17	1	0	0	
	Trentino Alto	1	1	NA	0	
	Combined	24	2	0.083	0.00014	−0.192
**Romania**	Bucharest	23	1	0	0	0
**Chile**	Santiago	31	2	0.065		−0.426
**Group A**		234	3	0.059	0.00045	−1.174*
**Group B**		39	5	0.63	0.00158	1.453

In the neutrality test, the Fu’s Fs statistic values were very low in the two native countries of BMSB, China and Japan, with − 7.852 (*p* < 0.02) and − 29.707 (*p* < 0.02) (Table 2) while for the BMSB-invaded countries, Fu’s Fs statistic value was − 1.174 (*p* < 0.02) for group A (Chile, Georgia, Hungary, Italy, Romania, Turkey and the USA) suggesting that group A was under population expansion. In comparison, the haplotype diversity was slightly higher with an average of 0.63 for group B (Austria, Serbia, and Slovenia), but with Fu’s Fs values of 1.453 (*p* > 10), indicating that group B was under bottleneck.

**Fig. 4:**
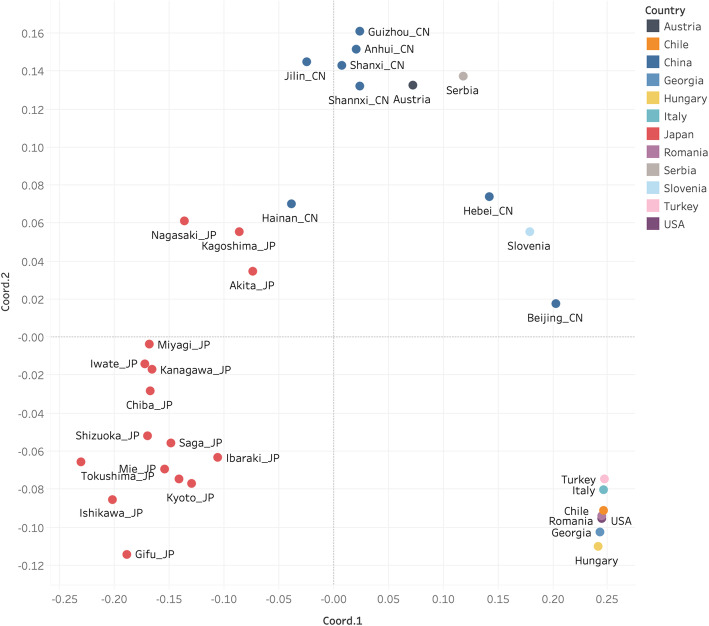
Principal Coordinates Analysis (PCoA) plot based on population pairwise genetic distances. The points from Austria, Chile, Georgia, Hungary, Italy, Romania, Serbia, Slovenia, Turkey and the USA represent the samples collected from one country while the points from China and Japan represent the samples collected from one province. The colour represents the countries where the samples were collected from. The provinces are labelled with name tags. The X axis is the value of Coord.1 while Y axis is the value of Coord.2. Percentage of variation explained by coord.1, Coord.2 was 43.39 and 12.99%, respectively. The figure was generated using Tableau 2019 (https://www.tableau.com/) [34]

The Principle Coordinates analysis (PCoA) using the F_ST_ values showed that there were at least three population clusters, namely China, Japan and group A (Chile, Georgia, Hungary, Italy, Romania, Turkey and the USA) (Fig. 4, Additional file 3). The recent invasion in Slovenia showed genetic similarities to those from Hebei and Beijing provinces of China (Fig. 4). The BMSB populations from Austria and Serbia were also closely related to the Chinese populations of Shanxi and Anhui. The population from the Chinese province of Hainan also showed close relationship with a population from the Japanese province of Akita.

The AMOVA (Analysis of molecular variance) showed that the genetic variation among the 12 populations contributed 71.26% while variation within population contributed 28.74%. The overall F_ST_ value was 0.71 (*p* < 0.05), indicating that the genetic variation among populations was high.

**Fig. 5:**
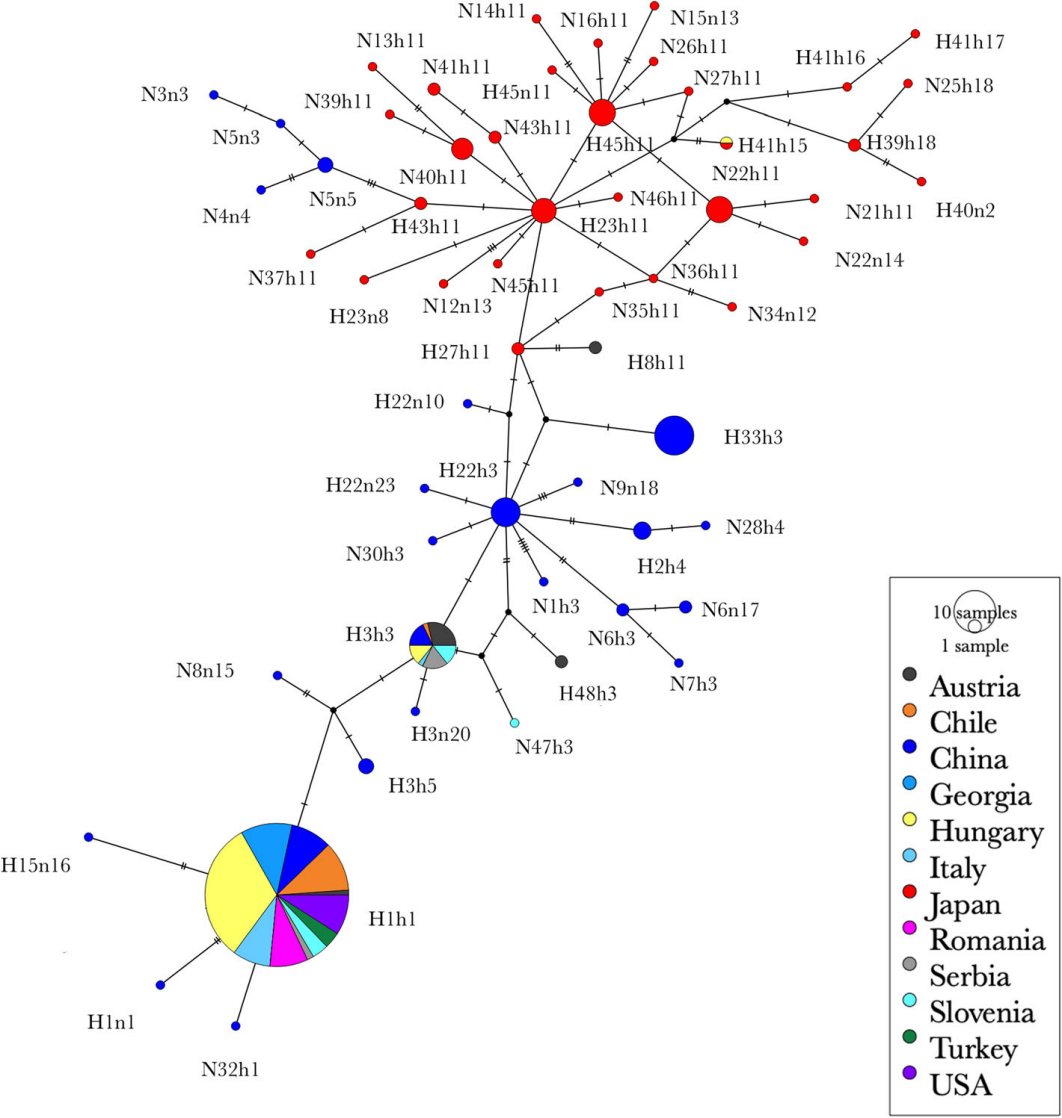
Haplotype network derived from the TCS analysis using COI-COII combined haplotypes (1175 bp fragment) of BMSB around the world. Each pie represents one haplotype with the haplotype name next to it. The size of pie is proportional to the frequency of haplotypes. The colour represents the countries where the samples were collected from. The tick marker on each line represents a base pair difference. The figure was constructed based on the combined COI and COII haplotypes obtained in the current study

**Fig. 6:**
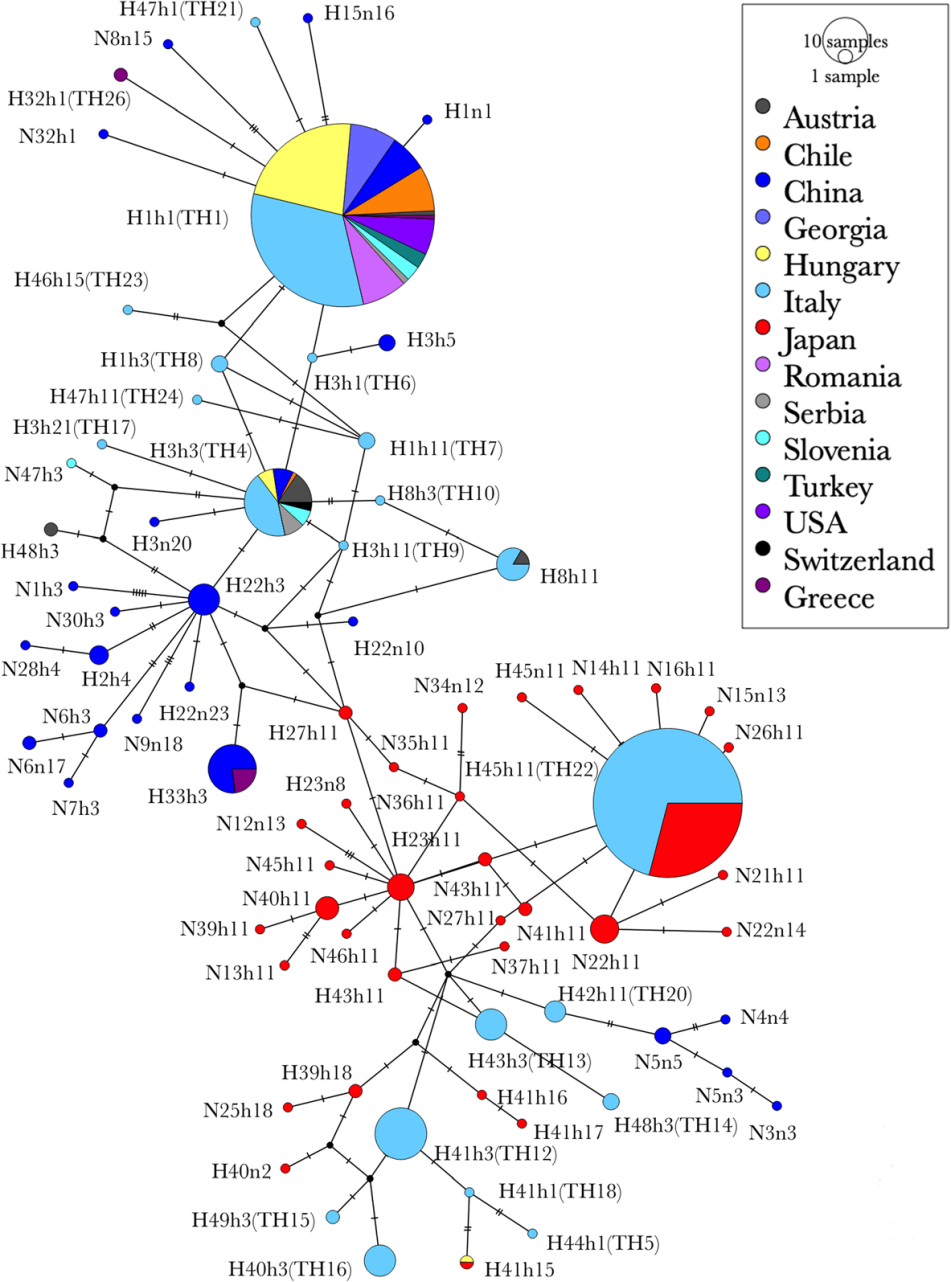
Haplotype network derived from the TCS analysis using COI-COII combined haplotypes (1175 bp fragment) of BMSB around the world. Each circle represents one kind of haplotype. The size of the circle represents the frequency of each haplotype. The colour represents the countries where the samples were collected. The tick marker on each line represents a base pair difference. The figure was constructed based on the haplotypes obtained in the current study and those from the study of Cesari et al. [30]. The haplotypes obtained from Cesari et al. [30] are labelled with TH

The haplotype network of the BMSB individuals further revealed the widespread occurrence of H1h1 and H3h3, except the populations from Japan, whereas all the other haplotypes were mainly detected in the native countries (Fig. 5). The analyses showed that there were three ancestral lines found in this study namely h1, h3 and h11. Most of the other haplotypes mutated from these three lines with differences of several base pairs. Moreover, an interesting phenomenon was observed that some haplotypes (N3n3, N5n3, N4n4, N5n5) detected only in the Hainan population (China) was highly isolated and closer to Japanese populations rather than to Chinese populations. To further explore the distribution of those haplotypes, the combined COI and COII dataset from the present and previous studies [20, 30] were analysed together and resulted in a total of 80 haplotypes. The haplotype network analysis (Fig. 6) indicated similar genetic relationships as previously reported except that a few BMSB specimens from Italy had close relationship with Japanese populations (Fig. 6).

## Discussion

This study revealed 51 COI haplotypes (36 novel) and 29 COII haplotypes (20 novel) from 463 BMSB individuals of 12 countries. However, most of these haplotypes (80%) were detected only once (Table 1 and Additional file 2), indicating that these new haplotypes are less abundant in the populations we studied. The haplotype analysis of mtDNA sequences of the BMSB populations from 12 countries provided genetic information for the identification of the pathways of invasion and the possible sources of origin.

In terms of haplotype distribution, the predominant COII haplotypes for Beijing (China), Shaanxi (China), Japan and the USA were h1 (67.8%), h3 (75%), h11 (81.1%) and h1 (100%). Xu et al. [29] made a similar conclusion that the major COII haplotypes for Beijing (China) and Shaanxi (China), and Japan and the USA were h1 (50%), h3 (100%), h11 (38%) and h1 (100%). Lee et al. [28] identified COI haplotype H1 as the main COI haplotype for China (68%), Hungary (98.8%), Italy (80%) and the USA (92.5%). Similarly, this current study identified haplotype H1 as the predominant one, accounting for 27.6% of the Chinese samples, 93.4% of the Hungarian samples, 95.8% of the Italian samples, and all samples from the USA. The percentage of each haplotype in those countries varies slightly between the current and previous studies, which is likely due to the differences in the sample size. On the other hand, some divergences were also observed between the current and previous studies. For example, COII haplotype h14 was identified as the predominant haplotype (33%) in the Japanese population in Xu’s study [29] but was not detected in this study (Additional file 2). Variations in the haplotype numbers and percentages between the current study and that of Cesari et al. [30] were also observed. Cesari et al. [30] reported a total of 26 COI and COII combined haplotypes for BMSB specimens, mainly from Italy [30]. In contrast, only five haplotypes [H1h1 (TH1), H3h3 (TH4), H33h3 (TH25), H8h11 (TH11) and H45h11 (TH22)] were detected in our study, thus, the total number of known BMSB COI and COII combined haplotypes (both known and novel) has increased to 80 known so far. Cesari et al. [30] included relatively large number of samples from Italy (209 samples from 10 regions) and identified 22 unique COI and COII combined haplotypes. Our study was unable to identify any additional COI and COII combined haplotypes beside the common ones (H1h1, H3h3) in Italy due to the small sample size (24 samples from 3 locations) studied. However, there were five shared haplotypes [H1h1 (TH1), H3h3 (TH4), H33h3 (TH25), H8h11 (TH11) and H45h11 (TH22)] among the samples studied here and by Cesari et al. [30]. Of these, the first two haplotypes were the two most common haplotypes. Interestingly, the last 3 haplotypes [H33h3 (TH25), H8h11 (TH11) and H45h11 (TH22)] detected in Italy by Cesari et al. [30] were also detected in this study from China, Austria, and Japan. After combining the haplotype data from the two studies, haplotype network (Fig. 6) revealed that the BMSB populations in Italy had genetic relatedness to Japanese populations, sharing the same haplotype H45h11 (TH22). It has been shown that the predominant haplotypes, such as H1h1 and H3h3, found in Italy were also widespread in China, the USA, and other European countries [20, 30]. Therefore, it can be hypothesised that the BMSB populations in Italy possibly have originated from Asia, which can be supported by the extensive, ongoing cross-border travel and trade between Asia and Italy. However, invasion from North America cannot be ruled out as H1h1 was found also in the USA. Furthermore, haplotype H8 was detected in Switzerland [13] in 2012, in France [31] and Northern Italy in 2013 [20], suggesting the possible invasion of BMSB to Italy was from Switzerland [30] based on the geographical proximity. The detection of the same haplotype of H8 in Austria in the current study raised the possibility that the invasion in Austria might have originated from the neighbouring countries of Switzerland or Italy as they share borders. However, the widespread distribution of H1 and H3 haplotypes in Austria (Table 1) opens the likelihood of invasion from China as well.

The combined data for COI and COII led to the observation of several ancestral lineages, including h1 (H1h1), h3 (H3h3, H22h3, H33h3) and h11 lines (H45h11, N22h11, N40 h11). The haplotype networks support that most of the less abundant haplotypes were possibly evolved from these lines.

The haplotype diversity of BMSB from the native regions is much higher than that of the invaded populations. The haplotype diversity (*Hd*) of BMSB populations from China and Japan was 0.858 and 0.942, respectively, which clearly indicates that the genetic diversity of these two populations was much higher than that of the most of the BMSB populations in the invaded countries (*Hd* < 0.182). This conclusion agreed with the observations from the study by Xu et al. [29]. Another interesting result from our study was the absence of shared haplotypes from the two neighbouring native countries of BMSB, China and Japan, which was congruent with previous studies [20, 29]. The haplotype network highlighted that China and Japan had their own haplotype clusters, suggesting that there is limited or no interbreeding, probably the geographical barriers of the strait restricted gene flow among populations. It is also possible that China and Japan have put in place a strict quarantine inspection for BMSB to prevent human-mediated transportation with current intensive trade activities. As a result, these BMSB populations could have been evolving independently. The Principal Coordinates Analysis also supported this conclusion, in which, most of the Japanese and Chinese populations were clustered by themselves except populations from regions of Akita (JP) and Hainan (CN). This phenomenon was also found in Zhu’s study [35] that the BMSB from Hainan and Japan were in the same clade.

The PCoA also revealed that the genetic group A (Chile, Georgia, Hungary, Italy, Romania, Turkey, and the USA) could have become a relatively independent genetic group. The neutrality test supported this, where the Fu’s Fs value of the genetic group A (− 1.174, *p* < 0.02) was negative as that of the ancestral Chinese (− 7.852, *p* < 0.02) and Japanese populations (− 29.707, *p* < 0.02), indicating that the populations belong to genetic group A could have been under population expansion stage [35]. The relatively low differences in F_ST_ values between the populations within the genetic group A indicate that these populations could have originated from the same ancestral line. The close genetic relatedness among the BMSB populations from Chile, Georgia, Hungary, Italy, Romania and Turkey with the USA populations suggests that the late detection in those European countries might have originated from the USA. This also aligns well with the past invasion history of BMSB, where BMSB was first detected in the USA in mid-90s, and then spread throughout the country. Ten years after establishing in the USA, BMSB was detected in Switzerland in 2007 [36], then spread in Europe [14–19, 37] and Chile [21] recently. This study further suggested the secondary invasion from the USA to the European countries such as Georgia, Hungary, Italy, Romania and Turkey, and to Chile. In contrast, the populations of the genetic group B (Austria, Serbia and Slovenia) were clustered with the Chinese populations, but were genetically distant from the group A, indicating that these populations in group B originated from a different pedigree line or genetic group. The neutrality test’s Fu’s Fs value of genetic group B was positive (1.453, *p* > 10), which is consistent with the more recent detection of BMSB in these locations as the positive Fs indicated that these populations were under a population bottleneck [38]. The haplotype diversity (*Hd*) and nucleotide diversity (*π*) of these three populations in group B were higher than 0.5 and 0.001, respectively, implying that these countries have been invaded multiple times by BMSB from different origins.

This study further revealed that the predominantly common haplotypes H1h1 and H3h3 exist in China and the invaded countries. The reasons for this need further investigation, though one possibility could be due to the dominant distribution of these haplotypes in Asia, and thus they have higher chances to be transported passively to other regions. It is not clear whether there is a possibility that these BMSB haplotypes can adapt more easily to new environments than other haplotypes. Since the COI and COII haplotype analyses are only based on the information from the female lineages, further study on the genomic level using high-throughput sequencing techniques might be able to provide more information on the genetic diversity of the BMSB populations and may help clarify the past invasion scenarios in the future.

## Conclusions

The present study has revealed genetic diversity among BMSB populations using combined COI and COII datasets and provided better understanding of their potential invasion pathways. The genetic diversity among the BMSB populations from the native regions was much higher than those from the BMSB-invaded countries. The haplotype analysis further indicated that the invasion of BMSB has occurred multiple times in the past, probably at least partially due to international trade and travel. BMSB populations from the invaded countries, such as Chile, Georgia, Hungary, Italy, Romania, Turkey, and the USA were genetically close, but well separated from the Chinese populations. However, the BMSB populations from Austria, Serbia and Slovenia were more closely related to the Chinese populations. The results indicated that some individuals of the recent invasions into Chile, Hungary, Georgia, Turkey, Romania and Italy potentially originated from the USA without ignoring the likely chances of possible invasions from China due to the presence of the ancestral predominant haplotype H1h1. Moreover, the BMSB populations from Austria, Serbia and Slovenia were possibly of recent invasions from China. In conclusion, we believe that the novel haplotype information and the genetic diversity among the global BMSB populations will lay down the foundation for future population genomic studies and could help in formulating effective BMSB management strategies. This study will also help in tracing the origin of BMSB intercepted at the border in those countries, such as New Zealand, where the species has not yet established.

## Methods

### Sample collection and DNA extraction

BMSB specimens were collected from 43 regions/provinces in 12 countries (Austria, Chile, China, Georgia, Hungary, Italy, Japan, Romania, Serbia, Slovenia, Turkey, and the USA). All BMSB specimens were stored in 95% ethanol at − 20 °C and the species identity was confirmed by morphological characteristics by MPI entomologists. Total genomic DNA was extracted from each individual specimen using QIAGEN DNeasy® Blood & Tissue Kit with QIAGEN RNase A (Qiagen, Valencia, CA, the USA). The DNA quality and purity were determined using NanoDrop™ (CA, the USA) and quantified using QuantiFluor™ (CA, the USA) (Additional file 1).

### Polymerase Chain Reaction (PCR) and sequencing

The genetic diversity of the BMSB populations was determined by analysing the mitochondrial DNA (mtDNA) cytochrome c oxidase subunit I (COI) and cytochrome c oxidase subunit II (COII). The two markers were chosen due to their fairly high variability and large number of sequences previously reported. Partial sequence of the COI (657 bp) and COII (518 bp) genes were amplified using the genomic DNA as a template. The LCO1490 (5′-GGTCAACAAATCATAAAGATATTGG-3′) and HCO2198 (5′-TAAACTTCAGGGTGACCAAAAAATCA-3′) [39] primer pairs were used for amplification of the COI region. Similarly, the HhalysCO2F2 (5′-TAACCCAAGATGCAAATTCT-3) and HhalysCO2R2 (5′-CCATATATAATTCCTGGACGA-3′) primer pairs were used for amplification of the COII region [29]. The PCR reagents (reaction volume of 20 μl) for COI and COII were the same except the primers, comprising of 4.4 μl sterile deionized water, 10.0 μl 2X GoTaq® Green Master Mix (Promega), 1.0 μl of 5 μM Forward primer, 1.0 μl of 5 μM Reserve primer, 0.6 μl of 50 mM MgCl2, 1.0 μl of 10 μg/μl BSA. Thermocycling conditions for both reactions comprised of an initial denaturation step of 94 °C for 5 mins, followed by 40 cycles of denaturation at 94 °C for 15 s, annealing at 50 °C for 30 s, and extension at 72 °C for 45 s, followed by a final extension phase at 72 °C for 7 min. All reactions were performed using a Veriti 96-well thermal cycler (Life Technologies). In this study, 463 specimens were used for COI and COII sequencing. The amplified DNA fragments in the final PCR product was evaluated on 1% agarose gel against a 100 bp DNA ladder (Invitrogen™) in TAE buffer stained with SYBR safe (Life Technologies) and visualised using a Gel Doc Software system (BioRad, Hercules, CA, the USA). The resulting product was diluted 5-fold with sterile water, and sent for Sanger sequencing Macrogen (Soul, South Korea) using the amplification primers in both directions. The quality of the Sanger sequencing dataset was manually examined and analysed in Geneious software (Biomatters, Auckland, New Zealand) [40]. The resulting quality-filtered COI and COII gene sequences for each haplotype were submitted to GenBank under the accession numbers, MT517228 - MT517274 for COI and MT490838 - MT490860 for COII. All the metadata for each specimen and their sequences were submitted into BOLD under project code BMSB. The BOLD processed IDs for each specimen are listed in Additional file 1. All the sequences were used to create a dataset in BOLD, under the DOI: dx.doi.org/10.5883/DS-BMSB.

### Public COI and COII sequence data acquisition and haplotype calling

A comparative barcoding analysis of COI and COII genes from the BMSB cohort of the current study and the publicly available COI and COII sequences from BMSB were conducted. The reference COI and COII sequences were created, respectively, by the following steps. Firstly, previously reported COI and COII barcode sequences of BMSB were downloaded from the GenBank. The COI (UID: 1674561291, 1,591,437,641, 1,334,761,755, 1,304,534,304, 1,240,496,350, 1,201,369,261, 1,024,298,892, 985,693,878, 443,298,673, 537,366,792, 552,099,040) and COII (UID: 1334762135, 552,098,974) sequences were aligned separately using Geneious software v10.2.5 [40]. Secondly, for COI, a 657 bp region was selected for further analysis while a 518 bp region was selected for COII. Sequences not from the same region of COI or COII were discarded. Finally, the aligned sequences were trimmed to 657 bp for COI and 518 for COII and duplicated sequences removed. The remaining unique sequences were used as the reference dataset for haplotype assignment.

All the COI and COII sequences obtained in this study were checked and edited in Geneious software [40] to remove poor quality sequences. A 657 bp COI and a 518 bp COII regions were used for further analysis, thus no missing data and ambiguous base-calls such as Y, W, N. S in the sequences obtained. Using the previously reported BMSB haplotypes as a reference, an in-house python script was developed to allocate the haplotype name for each obtained sequence. The script automatically allocated haplotype name to each individual of this study by searching the database. Sequences identical with those previously reported were assigned the same haplotype name (with a prefix H for COI or h for COII). The remaining sequences which did not share 100% sequence identity with the previously reported were given a new haplotype name (with a prefix N for COI and n for COII). Finally, a Microsoft Excel file was created (Additional file 1) and the number of haplotypes per population was calculated in Microsoft Excel. To further confirm those new haplotypes, all the sequences detected in this study were also BLAST searched in GenBank database [32]. To better illustrate the population genetics, the COI and COII sequences were concatenated into one linked longer haplotype, namely COI-COII combined haplotype. All the subsequent genetic analyses were conducted based on the COI-COII combined haplotypes.

### Population genetic analysis

The genetic diversity as the percentage of each haplotype present from different regions and/or countries was estimated by calculating the number of haplotypes detected at the country, divided by the total number of individuals sampled using Microsoft Excel. Population genetic diversity, as indexed by the number of haplotype (*Hn*), haplotype diversity (*Hd*) and nucleotide diversity (***π***) was estimated using DnaSP v6 [41] to quantify the degree of genetic diversity. Analyses of haplotype and nucleotide diversity were conducted separately for each population as well as for populations in one country and one genetic group. To examine the historical demographic expansion, a neutrality test was performed under DnaSP v6. Based on the *Hd* values, the BMSB populations in the invaded countries were divided into two groups: genetic group A (Chile, Georgia, Hungary, Italy, Romania, Turkey and the USA) and genetic group B (Austria, Serbia and Slovenia). Therefore, the neutrality test was also performed among the BMSB populations from China, Japan, group A, and group B.

Genetic differentiation among the BMSB populations was estimated by the fixation index (F_ST_), and the overall genetic variance was calculated by AMOVA (Analysis of molecular variance). Both calculations were fulfilled using Arlequin 3.5 [42]. To show the genetic relationships among the populations, a Principal Coordinates Analysis (PCoA) was conducted based on the F_ST_ data (Additional file 3) using GenAlEx 6.5 [43]. The relationships among the COI and COII combined haplotypes were examined using a parsimony network by applying the method described by TCS analysis [44] based on the COI-COII combined haplotypes and visualized using PopART [45].

## Supplementary Information

**Additional file 1.** Metadata information of the collected BMSB cohort.

**Additional file 2.** Haplotype information of the BMSB populations.

**Additional file 3.** F_ST_ values of all the BMSB populations.

## Data Availability

The in-house script can be found using the link below. https://1drv.ms/u/s!AvRFcQuxR5sIgpZzg_Ch02Lm2u4tJA?e=u9CoLx The dataset for the COI and COII sequences in BOLD can be found under the DOI: dx.doi.org/10.5883/DS-BMSB.

## Abbreviations

AMOVA: Analysis of molecular variance
BMSB: Brown marmorated stink bug
COI: Cytochrome c oxidase I
COII: Cytochrome c oxidase II
mtDNA: Mitochondrial DNA
MPI: Ministry for Primary Industries
NZIER: New Zealand Institute of Economic Research
PCoA: Principle Coordinates Analysis
